# Endoscopic ultrasound-guided transduodenal ERCP for hepatico-jejunostomy stricture

**DOI:** 10.1055/a-2218-2260

**Published:** 2024-01-09

**Authors:** Jun Liang Teh, Shannon M Chan, Hon Chi Yip, Anthony Yuen Bun Teoh

**Affiliations:** 1 Department of Surgery, Chinese University of Hong Kong, Hong Kong, Hong Kong; 2 Department of Surgery, Chinese University of Hong Kong, Hong Kong, Hong Kong

A 75-year-old man was referred for management of a hepaticojejunostomy (HJS) stricture following HJS performed for a bile duct injury during cholecystectomy 3 months prior. A short-type double-balloon enteroscopy (DBE) was attempted but was unsuccessful due to inability to reach the HJS. Endoscopic ultrasound (EUS)-guided transduodenal endoscopic retrograde cholangiopancreatography (ERCP) for management of the HJS stricture was planned 1 .

An EUS-guided duodenum–afferent limb bypass was first performed with a lumen-apposing metal stent (LAMS) between the duodenum and the afferent limb ( Video 1 ). On EUS, the afferent limb was identified from the duodenum and punctured with a 19G needle (EZshot 3; Olympus Medical, Tokyo, Japan) ( Fig. 1 ).The afferent limb was distended by infusion of 500 ml of normal saline mixed with indigo-carmine and contrast medium. Over a 0.025-inch guidewire, the delivery system of the cautery-enhanced LAMS delivery system (Hanarostent Z-EUS IT; M.I. Tech, Gyeonggi-do, South Korea) was inserted and a 16 × 20-mm stent was deployed into the afferent limb ( Fig. 2 , Fig. 3 ) 2 . ERCP was subsequently performed after 3 days with a dual-channel endoscope inserted into the afferent limb via the LAMS. The HJS ( Fig. 4 ) was dilated with a 6-mm biliary balloon (Hurricane Biliary RX; Boston Scientific, Marlborough, Massachusetts, USA). Two plastic stents were inserted into bilateral intrahepatic ducts.

Cannulation of the afferent limb via the lumen-apposing metal stent (white arrow) deployed between the duodenum and afferent limb bypass. Dilatation of the stenosed hepaticojejunostomy orifice was performed with a 6-mm biliary balloon.Video 1

**Fig. 1:**
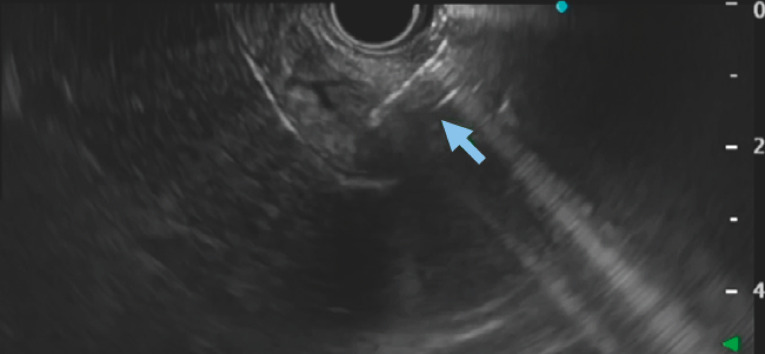
Identification of the afferent limb and puncture of the afferent limb with a 19G FNA needle.

**Fig. 2:**
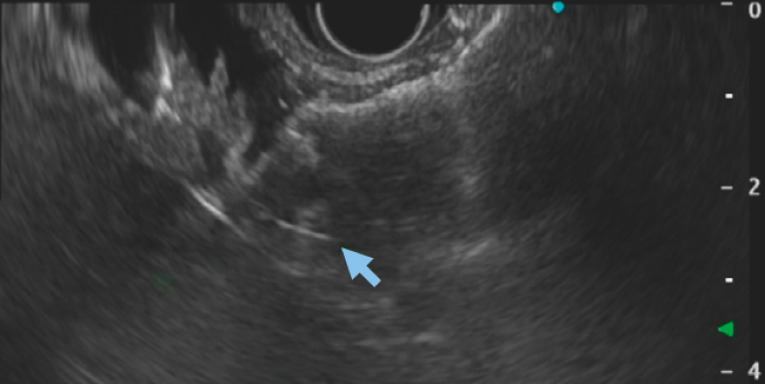
Deployment of the distal flange of the lumen-apposing metal stent (LAMS). The LAMS was pulled back before deployment in the channel and full deployment under endoscopic guidance.

**Fig. 3:**
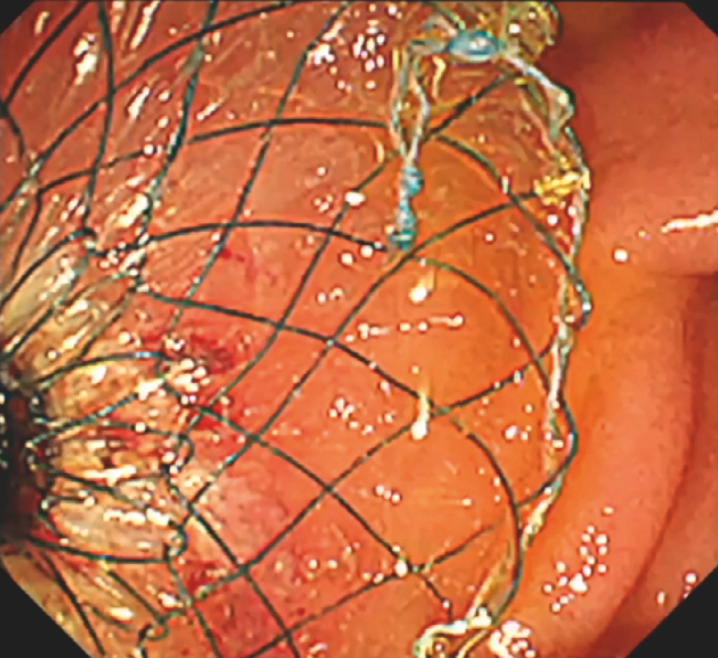
Endoscopic view of the fully deployed LAMS between the duodenum and the afferent limb.

**Fig. 4:**
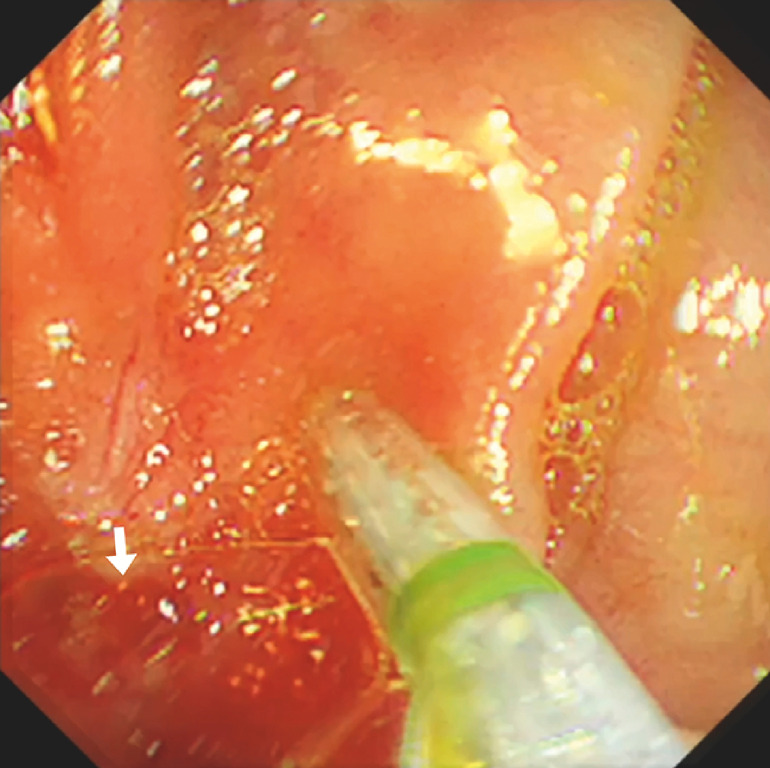
Identification of the stenosed hepatico-jejunostomy orifice (white arrow) in the afferent limb.

The patient was discharged on post-procedure day 2. No other complications or unplanned procedure occurred on follow-up. The patient underwent stent exchange at 6 months. No residual stricture was evident on cholangiogram at 9 months post-procedure. The HJS stricture occurs in up to 12.5% of patients at 2 years post hepatico-jejunostomy 3 . In this patient with an HJS stricture after Roux-en-Y HJS, EUS-guided duodenum–afferent limb bypass was successful for access to the HJS for ERCP after failed DBE-assisted ERCP.

Endoscopy_UCTN_Code_TTT_1AR_2AG

## Notes

Endoscopy E-Videos https://eref.thieme.de/e-videos
*E-Videos* is an open access online section of the journal *Endoscopy* , reporting on interesting cases and new techniques in gastroenterological endoscopy. All papers include a high-quality video and are published with a Creative Commons CC-BY license. Endoscopy E-Videos qualify for HINARI discounts and waivers and eligibility is automatically checked during the submission process. We grant 100% waivers to articles whose corresponding authors are based in Group A countries and 50% waivers to those who are based in Group B countries as classified by Research4Life (see: https://www.research4life.org/access/eligibility/ ). This section has its own submission website at https://mc.manuscriptcentral.com/e-videos .
